# The influences of target size and recent experience on the vigour of adjustments to ongoing movements

**DOI:** 10.1007/s00221-022-06325-7

**Published:** 2022-02-19

**Authors:** Eli Brenner, Hidde Hardon, Ryan Moesman, Emily M. Crowe, Jeroen B. J. Smeets

**Affiliations:** Department of Human Movement Sciences, Vrije Universiteit Amsterdam, van der Boechorststraat 7, 1081 BT Amsterdam, The Netherlands

**Keywords:** Online control, Interception, Movement time, Perturbation, Optimal control

## Abstract

People adjust their on-going movements to changes in the environment. It takes about 100 ms to respond to an abrupt change in a target’s position. Does the vigour of such responses depend on the extent to which responding is beneficial? We asked participants to tap on targets that jumped laterally once their finger started to move. In separate blocks of trials the target either remained at the new position so that it was beneficial to respond to the jump, or jumped back almost immediately so that it was disadvantageous to do so. We also varied the target’s size, because a smaller, less vigorous adjustment is enough to place the finger within a larger target. There was a systematic relationship between the vigour of the response and the remaining time until the tap: the shorter the remaining time the more vigorous the response. This relationship did not depend on the target’s size or whether or not the target jumped back. It was already known that the vigour of responses to target jumps depends on the magnitude of the jump and on the time available for adjusting the movement to that jump. We show that the vigour of the response is precisely tuned to the time available for making the required adjustment irrespective of whether responding in this manner is beneficial.

Goal-directed movements are under continuous control to ensure that they reach their target as precisely as possible (reviewed in Brenner and Smeets 2018). This is important for dealing with changes that occur during the movement, for instance because someone pushed your arm or a gust of wind moved the ball you were about to hit. It is known that movements are quickly adjusted in response to unexpected mechanical perturbations (Smeets et al. 1990) as well as to sudden displacements of the target of the action (Goodale et al. 1986), or even of nearby obstacles (Aivar et al. 2008). Such fast adjustments are made more or less automatically, even when they are not needed (Pisella et al. 2000; Voudouris et al. 2013). The vigour of fast responses to a target being displaced is known to depend on the magnitude of the displacement and on the time that is available for making the adjustment (Oostwoud Wijdenes et al. 2011; Zhang et al. 2018), but considering further factors might also help optimize the movement (Liu and Todorov 2007; Todorov 2004; Todorov and Jordan 2002).

We sought for evidence that adjustments are tailored to more factors than the magnitude of the target displacement and the time available for dealing with the displacement. In particular, we evaluated the influences of target size and recent experience in coping with target displacements. It is not necessary to adjust a movement to a large target as much as one to a small target, because a large target can be reached with a smaller adjustment (Knill et al. 2011). We therefore examined whether the response was less vigorous when the target was larger. Moreover, it makes sense to fine-tune the vigour of the response on the basis of recent experience. We therefore also investigated whether responses to a sudden displacement of the target of a goal-directed movement were less vigorous if on previous trials the target consistently moved back to its original path shortly after it was displaced. We compared responses to target displacements in blocks of trials in which the target moved back with responses to target displacements in blocks in which it did not. In the former case there was no need to correct the movement, whereas in the latter it was necessary to do so. By regularly switching between these two circumstances we hoped to get an idea of how quickly experience can influence the response to a sudden target displacement.

## Methods

### Participants and sessions

Ten naïve adults (one female, nine male) took part in the experiment after signing an informed consent form in accordance with the approval of the ethical review board. Participants took part in two sessions, each consisting of 12 blocks of 20 trials. Within each block the target jumped leftward on ten trials and rightward on the other ten trials, in a random order. In alternating blocks of trials, the target either remained on its laterally displaced path or jumped back to its original path after 150 ms. The first two blocks of trials within each session were not analysed. They were included to ensure that the remaining five pairs of blocks started with a similar history and also gave participants a chance to get accustomed to the task. All sessions started with a block in which the target remained on its laterally displaced path. Within each session all targets had the same size, but target size differed between the two sessions. The order of the sessions was counterbalanced across participants. There was a short break between the two sessions. Participants were not informed about the block structure of the sessions. Neither were they warned that the circumstances were about to change at the transitions between blocks.

### Equipment

Participants stood in front of a large screen (Techplex, 1.25 × 1 m; slanted 30° backwards) onto which images were back-projected at 120 Hz (800 × 600 pixels). The position of an infrared marker attached to the nail of the index finger of the participants’ preferred hand was tracked at 500 Hz using an Optotrak 3020 system. The marker’s position was related to positions in the image on the screen on the basis of a simple four-point calibration that was conducted before each session. To synchronize the timing of the marker positions with the timing of changes in the image, the Optotrak also recorded the position of a second marker that was attached to the side of the screen. This marker briefly stopped emitting infrared light so that its position was registered as ‘missing’ when a light-sensor registered a flash that was presented at the top left corner of the screen at the moment that the target jumped.

### Procedure

**Fig. 1:**
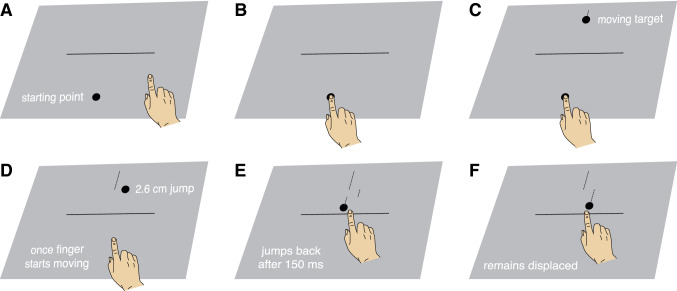
Schematic representation of a trial. **A** A starting point and a horizontal black line appear on the grey background. **B** Participants start the trial by placing their finger at the starting point. **C** After some time the starting point disappears and the moving target appears. **D** As soon as the participant lifts his or her finger the target jumps 2.6 cm laterally, either to the right (as shown) or to the left. **E** On half the trials the target jumps back to its original path. **F** On the other half it remains on the laterally displaced path

Each trial started with a 50 cm long horizontal black line and a starting point (a 1.5 cm diameter black disk) being presented on a 115 × 88.5 cm grey background (Fig. 1A). The starting point was presented at the centre of the lower part of the screen. When participants were ready for the trial, they placed their finger on this starting point (Fig. 1B). Once they had kept their finger at the starting point for a randomly chosen duration of between 600 and 1200 ms, a moving target appeared 48 cm above the starting point (Fig. 1C). The target was a black disk that could have a diameter of 3 cm (large target) or 1.5 cm (small target), and was always moving downward at 10 cm/s. The participants’ task was to tap on the target before it reached the horizontal line. The horizontal line’s vertical position was adjusted to the size of the target so that the target’s leading edge would reach the line after 800 ms (consequently, the line was 9.5 cm below the position at which the target appeared when the target was large, and 8.75 cm below the position at which the target appeared when the target was small).

As soon as participants lifted their finger more than 5 mm from the screen, the target jumped 2.6 cm to the left or to the right (Fig. 1D) while continuing to move downwards. In half of the blocks, the target jumped back to its original path after 150 ms (Fig. 1E). In the other half of the blocks it remained on the laterally displaced path (Fig. 1F). In all cases, it continued to move until it was tapped (or was not tapped within 500 ms of the finger starting to move, which was about when the target reached the line). A tap was characterized by the acceleration of the finger being more than 50 m/s^2^ in the direction away from the screen. If the marker on the finger was within the bounds of the target at the time of a tap, the finger was considered to have hit the target. If so, the target stopped moving and a sound indicated that the trial was successful. If the finger missed the target, the target moved away from the finger at 1 m/s.

### Analysis

To see how performance changed within blocks of trials, we divided the 20 trials of each block into ten pairs of two trials by combining the first trial with a leftward jump with the first trial with a rightward jump, the second trial with a leftward jump with the second trial with a rightward jump, and so on. We determined the measures of interest for each sequential pair of trials, and then determined the median values for each pair in the sequence across the 5 replications of each block for each participant. We present means and standard errors of the resulting values across the ten participants. As general measures of performance we examined the fraction of targets that were hit and the median time taken per tap. The time it took to tap was the time between when the target appeared and when a tap was detected.

We were mainly interested in the vigour of the response to the (first) target jump. The target jumped laterally, orthogonal to the direction from the starting point to where the target appeared and therefore to the finger’s main movement direction. The finger’s lateral movement was therefore almost exclusively determined by the response to the jump. To account for the fact that movements can be curved, irrespective of the target jumping, we compared the lateral movements in pairs of trials in which the target jumped leftward and rightward. Since the direction of the jump was randomized, participants could not predict the direction of the jump, so any systematic difference between lateral movements after leftward and rightward jumps must be a response to the jump.

We quantified the vigour of the response as the peak lateral acceleration of the finger in the direction of the target jump. To determine this value, we first determined the lateral acceleration of the finger at each moment from the first target jump. We did so with a second order Savitzky–Golay filter that was applied to all position samples within 20 ms of each measured sample: the fit coefficient of the quadratic term of a second order polynomial that was fit to a moving window of 21 measured values was assigned to the time of the central measured value. Having determined how the lateral acceleration changes with time for each trial, we then subtracted the lateral acceleration after the leftward jump from the lateral acceleration after the rightward jump within each pair of trials.

To visualize how the finger approached the target we determined the change in the finger’s lateral position as a function of the time before the tap. We determined the difference between the lateral positions of the finger for leftward and rightward target jumps, but halved the value so that the scale could be related to the size of the target. To relate the vigour of the response to the time available for adjusting the movement we determined the time from when the (first) jump occurred until the tap. We call this the *remaining time.* All analyses, except those comparing participants, were conducted separately for the large and small target and for each pair of trials in the sequence.

### Data

There were 4000 trials: 400 for each of the 10 participants. All these trials were considered when determining the fraction of targets that were hit. For all other measures there were trials that could not be considered. The finger did not move towards the target at all on 5 trials. Since this meant that the target never jumped, they could not be used to determine the *difference in lateral acceleration* after leftward and rightward target jumps or the *vigour of response.* As the analysis was based on pairs of trials this left us with 3990 of the 4000 trials for these measures. Measures that relied on the time of the tap were more problematic. On some trials the end of the movement was not detected during the experiment because the participant tapped too gently or rotated their finger (and thereby the infrared marker) away from the Optotrak cameras during the movement. Moreover, the finger marker recording stopped if the participant did not hit the target in time.

To determine how the lateral position of the finger depends on the *time before tap* we had to know the precise moment of the tap on every trial that we wanted to consider, so we had to exclude 450 trials (including the 5 in which the finger did not move at all), leaving us with 3550 trials. For the *time taken per tap* and the *remaining time* we only need to know the median value across the 5 replications, so rather than excluding trials for which the end of the movement was not detected we estimated the moment of the end of the movement for such trials. When participants tapped too gently they did not receive feedback about their tap, but the time of the tap could be estimated from the recordings: we used the moment the finger stopped moving towards the screen. For the remaining trials we used linear extrapolation of the final part of the finger’s movement to estimate the moment of the tap. If this estimate was less than 50 ms after the last valid marker recording we considered this estimate to be the moment of the tap. On 148 trials (including the 5 in which the finger did not move at all) the finger did not appear to have almost reached the screen when the recording ended or, on very few occasions, the marker was hidden from view for quite some time as it approached the screen. In these cases the *time taken per tap* and the *remaining time* were assigned extremely large values. Since we used median times in our analyses the precise value is irrelevant; these values were never the median value. We could determine the *difference in lateral acceleration* for all taps except those in which the finger did not move at all, because for this we only need to know the moment of the jump (not of the tap).

## Results

**Fig. 2:**
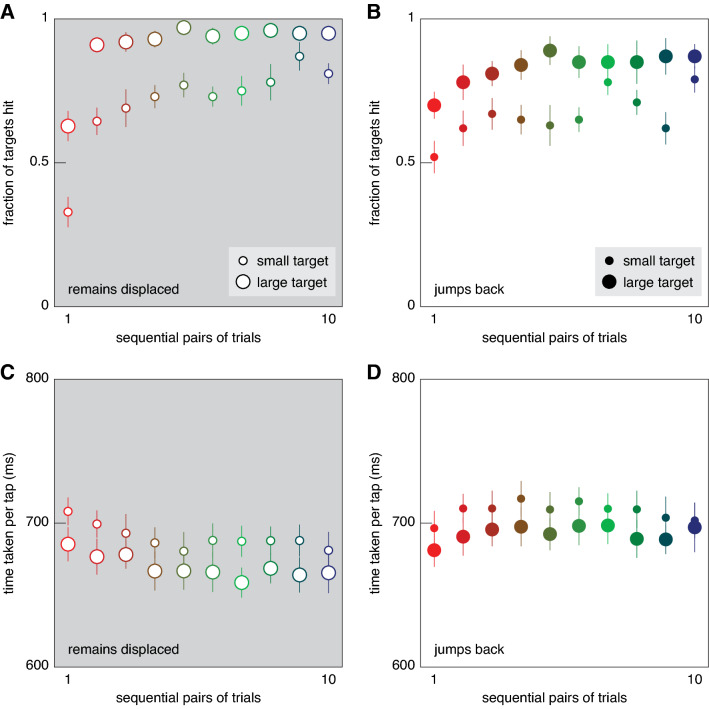
General measures of performance. Symbols indicate sequential pairs of trials within a block, where the first pair consists of the first leftward and first rightward jump, and so on. Target size is indicated by the size of the symbols. Error bars are standard errors across the ten participants’ values. The fraction of targets that were hit was larger for large targets, and increased during blocks of trials both when the target remained displaced (**A**) and when it jumped back to its original position (**B**). The time taken per tap (irrespective of whether the target was hit) was shorter for large targets. It decreased during blocks in which the target remained displaced (**C**) and increased in the blocks of trials in which the target jumped back to its original position (**D**)

Not surprisingly, participants hit more of the larger targets. They also took less time to do so (compare large and small symbols in Fig. 2). Participants hit more targets when the target remained displaced so that an adjustment was required (Fig. 2A), than when the target jumped back so that no adjustment was required (Fig. 2B). This was not because participants moved slower when they had to adjust the movement, because the time it took them to tap was shorter when the target remained displaced (Fig. 2C) than when the target jumped back (Fig. 2D). Both the fraction of targets hit and the time taken per tap changed systematically within each block. The fraction of targets that were hit increased during each block. The time taken per tap started near the final value of the previous block: the leftmost values in Fig. 2C are similar to the rightmost ones in Fig. 2D, and the leftmost values in Fig. 2D are similar to the rightmost ones in Fig. 2C. The time taken per tap gradually decreased when the target remained displaced, and it gradually increased when the target jumped back. The fraction of targets hit was lowest when the target first remained displaced after having jumped back on the preceding 20 trials (open red symbols in Fig. 2A), although participants moved quite slowly on such trials (high value of time taken per tap in Fig. 2C). The increase in the fraction of hits in the subsequent trials was accompanied by an increase in speed (a decrease in time taken per tap), so this is not just a shift in the speed-accuracy trade-off.

**Fig. 3:**
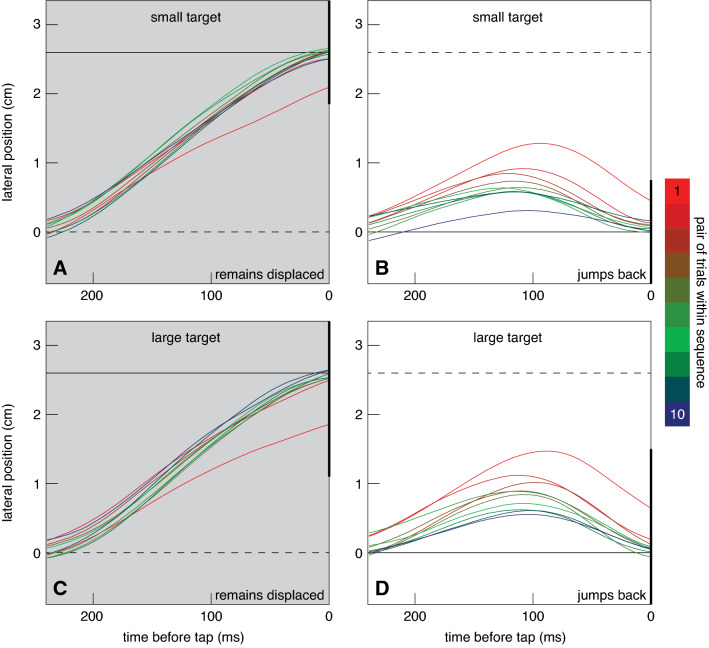
How target jumps influence the lateral position of the finger during the final part of the movement. Each curve shows half the mean difference between the finger’s lateral positions after the target jumped to the right and after it jumped to the left. We plot half the difference because this represents the change in position in response to a single 2.6 cm target jump. The change in lateral position is shown as a function of the time before the tap. Each curve represents a certain pair of trials in the sequence (colour coded). The background colour indicates whether the target jumped back. The thick vertical lines represent the target at its final position (note that the large target extends beyond the limits of the panels). The continuous horizontal lines represent the final position of the target centre. The dashed horizontal lines represent the initial position of the target centre (in **A** and **C**) or the position that the target jumped to before jumping back (in **B** and **D**)

The reason for fewer targets being hit at the beginning of a new block of trials is presumably that it takes participants a few trials to attune their response to the new circumstances. If it is the vigour of the response that changes, we might expect the finger not to move far enough to reach the target within the remaining time when the target unexpectedly remains displaced, and maybe to move so far in response to the first jump that it can no longer reach the original position within the remaining time when the target unexpectedly jumps back after 150 ms. To judge whether this is why participants missed many targets at the beginnings of new blocks, we plotted the lateral position of the hand as it approached the target. During the first pair of trials in which the target remained displaced, participants systematically adjusted too little, failing to fully account for the target jump (red curves in Fig. 3A, C) During the first pair of trials in which the target jumped back they responded too strongly to the first jump or too little to the jump back (or both; red curves in Fig. 3B, D). By the second pair of trials the finger ends close to the centre of the target. During the blocks of trials in which the target jumps back, the amplitude of the finger’s deviation appears to gradually decline as the sequence progresses (Fig. 3B, D). Thus, there is some indication that the vigour of the response is adjusted to the circumstances.

**Fig. 4:**
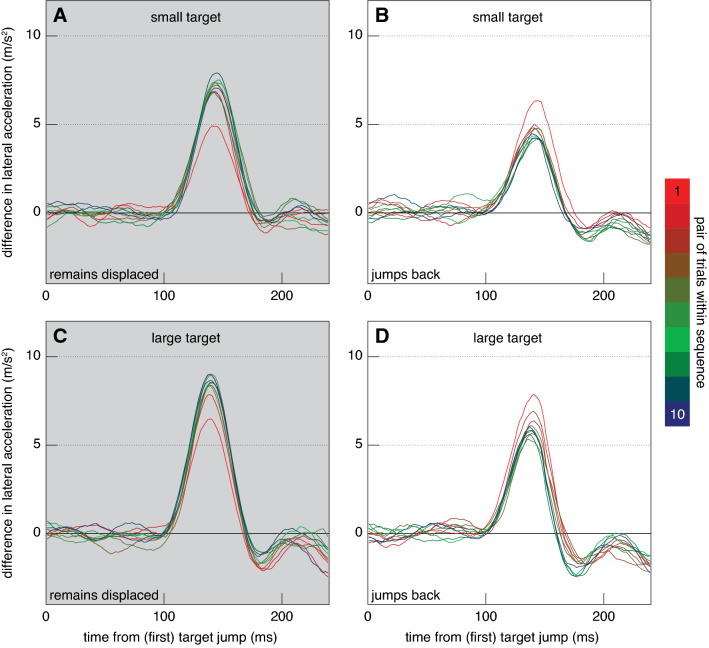
Responses to the (first) target jump. Each curve shows the mean difference between the lateral acceleration of the finger when the target jumped to the right and when it jumped to the left for a certain pair of trials in the sequence (colour coded). The background colour indicates whether the target jumped back. Responses are generally less vigorous when the target jumps back (**B**, **D**), except for the first trials of each block (red). They are also less vigorous for the small target (**A**, **B**) than for the large one (**C**, **D**)

**Fig. 5:**
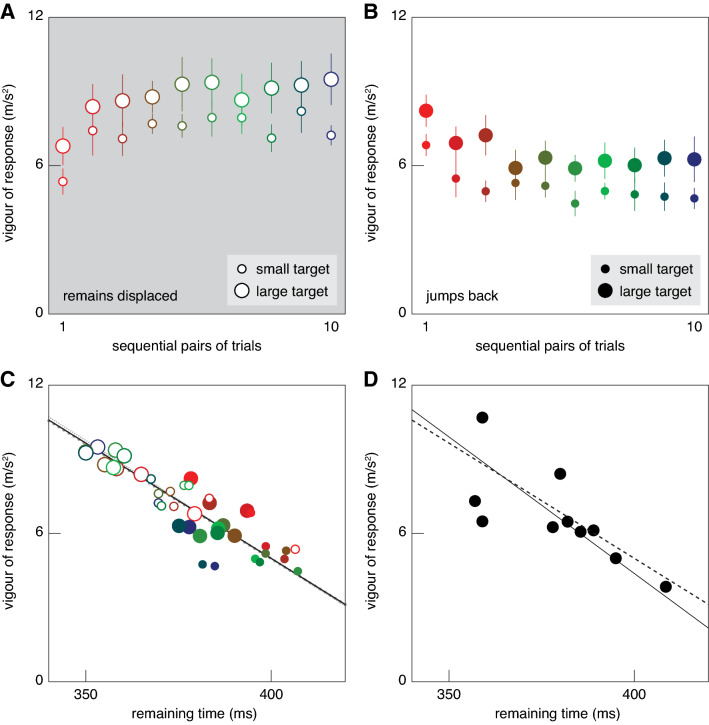
The vigour of the response. **A** The vigour quickly increased when the target stopped jumping back. **B** It quickly decreased when the target started jumping back again. Error bars are standard errors across the ten participants’ values. **C** The vigour clearly depended on the time between jump and tap. Symbols correspond to those in Panels A and B. The lines are fits to the data (see text). Doing the fit separately for large and small targets (dotted grey lines with large and small dots, respectively) gave very similar relationships to doing so for all the data (solid black line). **D** The same analysis as in **C**, but here each symbol shows a participant’s overall mean value rather than a different kind of target. The solid line is a fit to these symbols. The dashed line is the overall fit from **C**

To test the idea that response vigour is adjusted to the circumstances more directly, we turn to the lateral acceleration of the hand as a function of the time from the jump. The way in which the lateral acceleration changed during the two kinds of blocks was more or less consistent with the response vigour being adjusted to the circumstances. In the first trials in which the target remained displaced the response was less vigorous than on subsequent trials, irrespective of the target size (the bright red traces in Fig. 4A, C peak at a lower difference in acceleration). In the first trials in which the target jumped back the response was more vigorous than on subsequent trials (bright red traces in Fig. 4B, D). After the first pair of trials, the response was clearly less vigorous when the target jumped back than when it did not, which is consistent with the idea that the vigour of the response is regulated on the basis of the expectation that the target will behave as it had in the recent past. The vigour of the response quickly increased when the target stopped jumping back (Fig. 5A) and decreased once it started doing so again (Fig. 5B).

Contrary to our expectation, the responses were more vigorous for the larger target. When a response was required because the target remained displaced, the peak difference in lateral acceleration was higher for the large target (Fig. 4C) than for the small one (Fig. 4A). When no response was required because the target jumped back, the peak difference in lateral acceleration was also higher for the large target (Fig. 4D) than for the small one (Fig. 4B). Not finding less vigorous responses for the larger target is understandable on the basis of Fig. 3, because participants did not adjust the movement to a lesser extent when the target was larger. In all cases, they appeared to adjust their movements such that they would reach the target centre. Finding more vigorous responses for the larger target is consistent with the time taken to tap being shorter for the larger target (Fig. 2C, D). With less time left to make the necessary adjustments, the adjustments need to be more vigorous. It is known that the response vigour to step changes in target position is larger when there is less time available to make the necessary changes (Liu and Todorov 2007; Oostwoud Wijdenes et al. 2011; Zhang et al. 2018). Could such a change in timing account for all the changes in response vigour in Fig. 5A, B?

Plotting the vigour of the responses as a function of the remaining time (Fig. 5C), rather than as a function of the position within the block (Fig. 5A, B) reveals that the response is clearly less vigorous when there is more time. A systematic relationship between vigour of response and remaining time can account for the differences that we observed between blocks of trials with targets of different sizes (large and small symbols) as well as blocks of trials in which the target did or did not jump back to its original path (filled and open symbols). This relationship also captures the changes within the blocks (different colours of the same symbol): the particularly low vigour of responses for the first pair of targets that remained displaced was accompanied by a correspondingly long remaining time (bright red open symbols). Figure 5D shows that the same systematic relationship between vigour of response and remaining time is also evident when comparing performance across participants.

If response vigour is modified to perform the required adjustment within the remaining time, the actual relationship between the two must be curvilinear. However, the range of measured values for remaining time is limited, so we approximated the relationship within this range by fitting a straight line to the data (Fig. 5C). Since there is uncertainty in both variables, we used an orthogonal fit that minimizes the sum of the squared distances to the symbols, rather than a standard linear regression. Since the two measures have different units, we expressed the distances in terms of the corresponding standard deviations for doing the fit. A single linear relationship describes all the data quite well (solid black line). Performing the fit separately for the small and large targets (grey dotted lines) yields very similar results, so target size does not affect response vigour directly. The only tendency that is not explained by this relationship is that the responses to targets that will jump back (solid symbols) depend to some extent on their position in the sequence: early responses (red) are more vigorous than late ones (blue).

## Discussion

Our goal was to determine whether target size and recent experience with target jumps influence the vigour of adjustments to goal-directed movements. To achieve this, we had participants place their finger at a starting point at the bottom of a screen and then intercept targets that moved downwards towards that position from the top of the screen. They had to tap on the target before it reached a horizontal line. Once the finger left the starting point the target jumped to the left or to the right. We compared responses to identical jumps of large and small targets in blocks of trials in which the target either remained displaced, or jumped back very soon after its first jump. A smaller and therefore less vigorous adjustment would have sufficed to hit larger targets, because there was no need to hit the centre of the target. However, we found more vigorous responses for larger targets. When the target remained displaced, it was beneficial to respond to the jump. When the target jumped back, it was disadvantageous to respond to the first jump. We found that, after the first trials of each block, responses were indeed more vigorous when the target remained displaced than when it jumped back. However, all the observed changes in response vigour could be explained by the response being more vigorous when there was less time left in which to make the required adjustments (Fig. 5C, D). Thus, it seems that we can conclude that the vigour of adjustments only depends on the magnitude of the jump and on the remaining time to make the adjustment. The similarity between the dotted lines in Fig. 5C shows that the relationship between response vigour and remaining time does not depend on the target size, even if each measure on its own clearly does (for larger targets the response is more vigorous and the tap takes place sooner). That the same relationship between response vigour and remaining time is observed when comparing participants rather than experimental circumstances (Fig. 5D) supports the idea that the vigour of the response is primarily determined by this relationship.

The vigour of the response appears to be slightly higher during the first than during the last trials of blocks in which the target jumped back, even when differences in the time that elapsed between the target jump and the tap are taken into account (solid red and blue symbols tend to be above and below the line, respectively, in Fig. 5C). This might be a coincidence, but even if the response is slightly more vigorous during the first trials, we need not conclude that other factors than the magnitude of the target jump and the remaining time influence response vigour. A small apparent influence is easily explained by nuancing the idea that the vigour of adjustments is determined by the remaining time. On the first trial in which the target jumps back the participant does not know that it will do so, so the vigour of the response to the first jump is presumably determined by the expected movement time under the assumption that the target will remain at its new position. When the target jumps back the duration of the movement is longer (Fig. 2D). The increase in duration must be a response to the second displacement, so it cannot influence the response to the first jump, making the response to the first jump more vigorous than is appropriate for the remaining time as determined using the actual time of the tap. Thus, it is not that the response is exceptionally vigorous, but that the remaining time is underestimated. Following this reasoning, the solid red symbols in Fig. 5C are further to the right than they should be, so the fit line is slightly too shallow. Correcting for this would give a line that is even closer to the one for the differences between participants (Fig. 5D). We conclude that the vigour of the adjustment is determined by the expected remaining time at the moment at which the response is initiated.

**Fig. 6:**
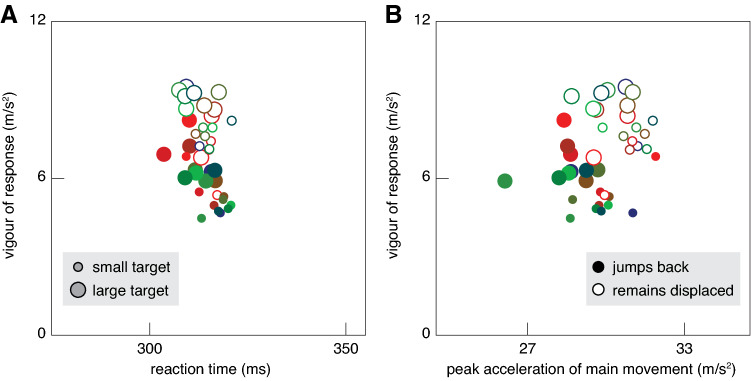
Alternative measures correlate less well with the vigour of the response. **A** Vigour of the lateral response as a function of the reaction time. **B** Vigour of the lateral response as a function of the peak acceleration in the main movement direction

To confirm that the (expected) remaining time determines the vigour of the response, rather than both the time taken to tap the screen and the vigour of the lateral response being determined by a general tendency to act faster under some circumstances, we conducted two additional post-hoc analyses. First, we examined the relationship between the vigour of the lateral response and the reaction time. As our measure of reaction time we used the time until the first target jump, so this value includes the time it took to move the finger 5 mm from the screen and the delay between the movement being detected and the displaced target being presented on the screen. The vigour of the response does not evidently depend on the reaction time (Fig. 6A). Since the target reaches the horizontal line 800 ms after the reaction time, this analysis also confirms that it is the time until the tap rather than the time until the target reaches the horizontal line that determines the vigour of the response. Secondly, we examined the relationship between the vigour of the lateral response and the peak acceleration in the main movement direction (upwards along the screen). The lateral response is orthogonal to the main movement direction, so the two accelerations are not automatically related, but it is conceivable that the lateral acceleration would be scaled to the acceleration in the main movement direction. However, this does not seem to be the case (Fig. 6B). Thus, we are confident that it is really the remaining time that is considered.

In trials in which the target jumped back there were two target jumps to respond to. We only analysed the vigour of the response to the first target jump, because our goal was to compare the response to the same target jump across blocks of trials in which the target remained displaced or jumped back. We saw that participants did not stop responding to the first target jump when exposed to targets repeatedly jumping back (Fig. 4B, D). We also saw that the finger ended up near the centre of the target on most trials in which the target jumped back (Fig. 3B, D). When the target jumped back there was therefore obviously also a response in the opposite direction that corrected for the finger’s lateral response to the initial target jump. The reason that this second response is not visible in Fig. 4 is that it started about 100 ms after the target jumped back, so about 250 ms after the first target jump (because the target jumped back after 150 ms), which is just beyond the time scale of Fig. 4. The movement time was longer when the participant expected the target to jump back (Fig. 2C, D). This is probably not only because the movement slowed down when the finger responded to the target jumping back, because the peak acceleration in the main movement direction already seems to be lower when the target jumped back (solid symbols further left than open symbols in Fig. 6C). Thus, the movement time was adjusted to the fact that the target jumped back, despite the fact that not responding to the jump would probably have made it possible to move faster.

Franklin and Wolpert (2008) also conducted a study comparing response vigour when it was either advantageous or disadvantageous to respond. In their study, participants moved a cursor to a static target. Participants could not see their hand, but saw a cursor that followed their unseen hand. In one condition the cursor shifted away from the hand so that a response was required for the cursor to reach the target. In another condition the cursor initially shifted away from the hand but then shifted back so that any response to the initial shift made it necessary to later move back in the opposite direction. Contrary to our findings, Franklin and Wolpert (2008) found much less vigorous responses when it had been disadvantageous to adjust the movement on preceding trials. An obvious difference between the two studies is that we shifted the target whereas they shifted a cursor representing the hand. Although these manipulations may seem equivalent, the responses to the two kinds of shifts are known to differ (Brenner and Smeets 2003). Responses to shifting a cursor may be more malleable because one can rely on haptic as well as visual information about the position of one’s hand. Just as haptic feedback at the end of a movement can influence the relative weights given to two conflicting visual cues when they are combined (Ernst et al. 2000; van Beers et al. 2011), the weight given to visual information about the moving hand might be reduced when it is advantageous to ignore such information. Such reduced weight would result in a less vigorous response to perturbation of the cursor’s position. Since vision provides the only source of information about a target’s position one cannot reduce the response to perturbation of a target’s position by relying on other cues. Another potentially relevant difference between the studies is that the target was moving in our experiment, so that participants could not simply reproduce the previous movement when the target jumped back to its original trajectory. Yet another potentially relevant difference is that participants had to move much faster in the present study (movement times of about 400 ms rather than about 700 ms), which may have prevented them from exploring better strategies. It has even been suggested that different mechanisms are recruited to guide the hand when the response has to be made quickly and the target is moving (Kozak and Corneil 2021).

Several studies have previously reported failures to suppress responses when doing so was disadvantageous (Aivar et al. 2008) or when specifically instructed not to respond (Pisella et al. 2000). In those studies participants could judge from the stimulus itself that they should not respond, rather than having to rely on recent experience. The failure to suppress responses under such circumstances is probably due to the fact that it takes longer to initiate the adequate action, even if that action is not to respond, than to divert the ongoing movement (Smeets et al. 2016). Here we examine whether recent experience with it being advantageous or disadvantageous to respond vigorously influences the vigour of the response. To gather a lot of data near transitions between blocks of trials in which it was either advantageous or disadvantageous to respond, we used blocks of 20 trials, so we only consider quite recent experience. Franklin and Wolpert (2008) used blocks of more than 200 trials in which it was either advantageous or disadvantageous to respond, preceded by blocks without perturbations. Their participants stopped responding to the cursor shifts within a few trials when it was disadvantageous to respond, so the number of trials is unlikely to be critical.

A comprehensive way to summarise our results is that the movement time is adjusted to the circumstances, and the vigour of adjustments is determined by an estimate of the remaining time at the moment of the target jump. We consider this to be a better description than that the vigour of the response is planned and the timing of the tap is adjusted to match the vigour, because the movement time is known to depend on factors such as the target’s size and distance (Fitts 1954) and is adjusted on the basis of recent performance (Brenner and Smeets 2011). Moreover, movement time is often restricted by the task. For instance, in the current study the target had to be hit before it reached the horizontal line. Only responding to the magnitude of the required adjustment and the remaining time makes sense if one considers responses to target jumps to result from a mechanism that normally guides ongoing movements to their target despite substantial sensorimotor variability, rather than as a special mechanism for dealing with unpredictable target jumps (Brenner and Smeets 2018). A similar case for responses resulting from a mechanism that normally guides ongoing movements to their target has been made for responses to mechanical perturbations (Crevecoeur et al. 2012), for which the vigour of fast responses also depends on the remaining time (Crevecoeur et al. 2013). The strong correlation that we found between the vigour of responses and the remaining movement time might mean that the vigour of the response is adjusted to the remaining time when the target jumps, but it is also possible that the two are both constantly adjusted together to optimize performance (Todorov and Jordan 2002).

## Conclusion

The vigour of online adjustments to target jumps is clearly related to the amount of time that one expects to be available for making the adjustment. Since the movement time is attuned to the circumstances, the vigour of adjustments is as well. We found no evidence that the vigour of the response is also independently influenced by circumstances that make a vigorous response more or less beneficial.

## Data Availability

The data and analysis scripts for the current study are available at https://osf.io/6hjxq/?view_only=55e0a3a6de544a4cb9eaec58179845ed

## References

[CR1] Aivar MP, Brenner E, Smeets JBJ (2008). Avoiding moving obstacles. Exp Brain Res.

[CR2] Brenner E, Smeets JBJ (2003). Fast corrections of movements with a computer mouse. Spat vis.

[CR3] Brenner E, Smeets JBJ (2011). Quickly 'learning' to move optimally. Exp Brain Res.

[CR4] Brenner E, Smeets JBJ (2018). Continuously updating one's predictions underlies successful interception. J Neurophysiol.

[CR5] Crevecoeur F, Kurtzer I, Scott SH (2012). Fast corrective responses are evoked by perturbations approaching the natural variability of posture and movement tasks. J Neurophysiol.

[CR6] Crevecoeur F, Kurtzer I, Bourke T, Scott SH (2013). Feedback responses rapidly scale with the urgency to correct for external perturbations. J Neurophysiol.

[CR7] Ernst MO, Banks MS, Bülthoff HH (2000). Touch can change visual slant perception. Nat Neurosci.

[CR8] Fitts PM (1954). The information capacity of the human motor system in controlling the amplitude of movement. J Exp Psychol.

[CR9] Franklin DW, Wolpert DM (2008). Specificity of reflex adaptation for task-relevant variability. J Neurosci.

[CR10] Goodale MA, Pelisson D, Prablanc C (1986). Large adjustments in visually guided reaching do not depend on vision of the hand or perception of target displacement. Nature.

[CR11] Knill DC, Bondada A, Chhabra M (2011). Flexible, task-dependent use of sensory feedback to control hand movements. J Neurosci.

[CR12] Kozak RA, Corneil BD (2021). High-contrast, moving targets in an emerging target paradigm promote fast visuomotor responses during visually guided reaching. J Neurophysiol.

[CR13] Liu D, Todorov E (2007). Evidence for the flexible sensorimotor strategies predicted by optimal feedback control. J Neurosci.

[CR14] Oostwoud Wijdenes L, Brenner E, Smeets JBJ (2011). Fast and fine-tuned corrections when the target of a hand movement is displaced. Exp Brain Res.

[CR15] Pisella L, Gréa H, Tilikete C, Vighetto A, Desmurget M, Rode G, Boisson D, Rossetti Y (2000). An ‘automatic pilot’ for the hand in human posterior parietal cortex: toward reinterpreting optic ataxia. Nat Neurosci.

[CR16] Scott SH (2016). A functional taxonomy of bottom-up sensory feedback processing for motor actions. Trends Neurosci.

[CR17] Smeets JBJ, Erkelens CJ, Denier van der Gon JJ (1990). Adjustments of fast goal-directed movements in response to an unexpected inertial load. Exp Brain Res.

[CR18] Smeets JBJ, Oostwoud Wijdenes L, Brenner E (2016). Movement adjustments have short latencies because there is no need to detect anything. Mot Control.

[CR19] Todorov E (2004). Optimality principles in sensorimotor control. Nat Neurosci.

[CR20] Todorov E, Jordan MI (2002). Optimal feedback control as a theory of motor coordination. Nat Neurosci.

[CR21] van Beers RJ, van Mierlo CM, Smeets JB, Brenner E (2011). Reweighting visual cues by touch. J vis.

[CR22] Voudouris D, Smeets JBJ, Brenner E (2013). Ultra-fast selection of grasping points. J Neurophysiol.

[CR23] Zhang Y, Brenner E, Duysens J, Verschueren S, Smeets JBJ (2018). Effects of aging on postural responses to visual perturbations during fast pointing. Front Aging Neurosci.

